# Tuberculous Osteomyelitis of the Scapular Spine Revealing HIV-1 Infection

**DOI:** 10.3390/tropicalmed10010008

**Published:** 2024-12-30

**Authors:** Khaoula Idsaid, Malika Idalene, Khadija Danaoui, Wiam Ait Driss, Rania Elfargani, Nabila Soraa, Noura Tassi

**Affiliations:** 1 Department of Infectious Diseases, University Hospital Mohamed VI, Marrakesh, Faculty of Medicine and Pharmacy FMPM, Cadi Ayyad University, Marrakesh 40000, Morocco; 2 Microbiology Laboratory, University Hospital Mohamed VI, Marrakesh 40000, Morocco

**Keywords:** osteoarticular tuberculosis, mycobacterium tuberculosis, tuberculous osteomyelitis, scapular spine, HIV-1

## Abstract

Tuberculosis is no longer confined to developing nations; it persists as a significant contributor to illness and death on a global scale. The subtle clinical manifestation and association with human immunodeficiency virus infection poses obstacles for early diagnosis and management. Tuberculosis manifesting at extrapulmonary sites is relatively rare. We herein present the case of a 26-year-old patient from Cameroon with a history of pleuropulmonary tuberculosis treated in 2008, who had been taking pre-exposure prophylaxis (PrEP). The patient presented with right shoulder pain of an inflammatory type. The case was diagnosed as tuberculous osteomyelitis of the scapular spine, complicated by a deltoid abscess. Diagnosis was confirmed using computed tomography and the MTB/RIF GeneXpert test on the abscess puncture. This rare form of tuberculosis with an exceptional site revealed a HIV infection with profound immunosuppression. The patient was initiated on anti-tubercular treatment according to Moroccan recommendations.

## 1. Introduction

Tuberculosis (TB) is a contagious infectious disease of human-to-human transmission caused by the pathogenic effects of a mycobacterium of the tuberculosis complex (mainly Mycobacterium tuberculosis hominis, also known as Koch’s bacillus (BK), less commonly Mycobacterium Bovis and Mycobacterium africanum) in the body [1].

Osteoarticular tuberculosis (OAT) is defined as all pathological manifestations resulting from Koch’s bacillus (BK) damage to osteoarticular structures of the musculoskeletal system [2].

With a prevalence ranging from 0 to 4%, osteoarticular damage of infectious cause is rare in HIV-1 infection [3]. Most cases reported in the literature are in severely immunocompromised patients (CD4 lymphocyte count < 50/mm^3^) [3].

HIV–tuberculosis coinfection is a global public health concern. According to the World Health Organization (WHO), one-third of people living with HIV worldwide are coinfected with the tuberculosis bacillus, particularly in countries where the endemicity of tuberculosis coincides with the spread of HIV. HIV infection increases the risk of TB infection progressing to TB disease and also increases the rate of progression of recent or latent infection [4]. Tuberculosis often presents with unusual clinical or radiological features, making its diagnosis in the presence of HIV infection difficult.

HIV-associated tuberculosis can be difficult to diagnose. Its clinical or radiological presentation is often atypical. Tuberculin skin tests often show anergy, and microscopic examinations of sputa are negative in most cases. Tuberculosis often presents as a generalized disease with extrapulmonary sites. These difficulties underscore the lower value of symptoms suggestive of tuberculosis in patients with HIV [5].

We report the case of a patient who presented with osteomyelitis of the right scapular spine revealing HIV-1 infection.

## 2. Case Presentation

A 26-year-old man from Cameroon with a history of pleuropulmonary tuberculosis treated and reported cured in 2008, who had been taking pre-exposure prophylaxis (PrEP) based on TDF (tenofovir disoproxil fumarate)-emtricitabine irregularly for more than 2 months before his admission, was admitted to the Infectious Diseases Department in Mohamed VI university hospital center of Marrakech because of right shoulder pain of an inflammatory type. The pain had developed progressively since 1 month before his admission, in the context of fever and severe deterioration of his general condition.

On examination, the patient was conscious with a Glasgow score of 15/15, blood pressure of 110/60, heart rate of 110 bpm, polypnea at 30 cpm with 99% room air saturation and a fever of 41 °C.

**Figure 1:**
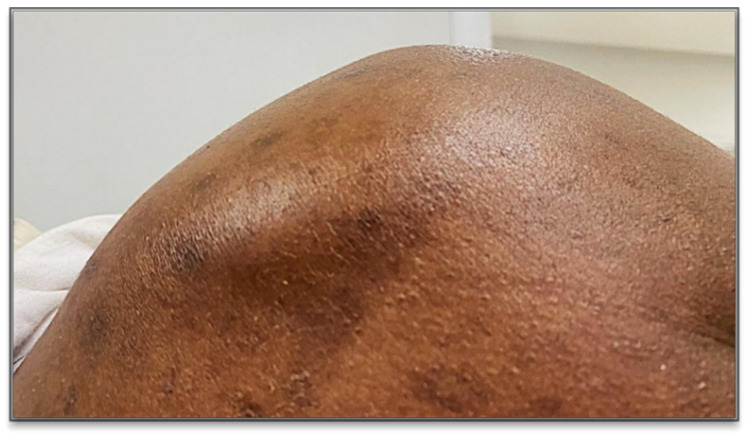
Appearance of the right shoulder.

**Figure 2:**
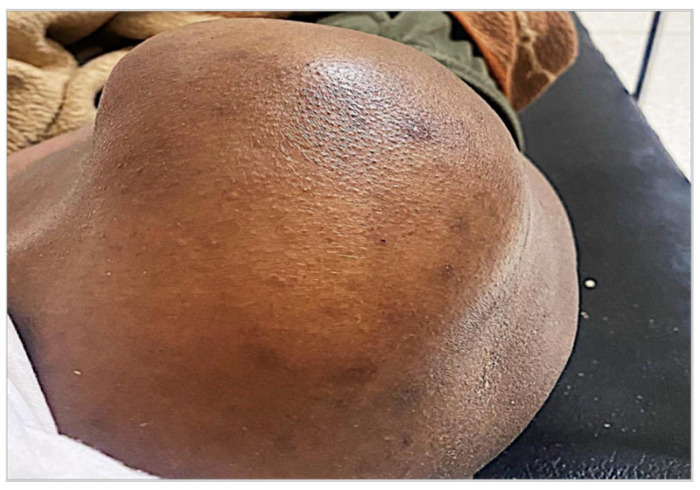
Swollen appearance of the right shoulder on examination.

Clinical inspection revealed a red, hot, painful joint with fluctuating swelling without tingling (Figure 1 and Figure 2). Active and passive mobilization of the right shoulder was impossible.

**Figure 3:**
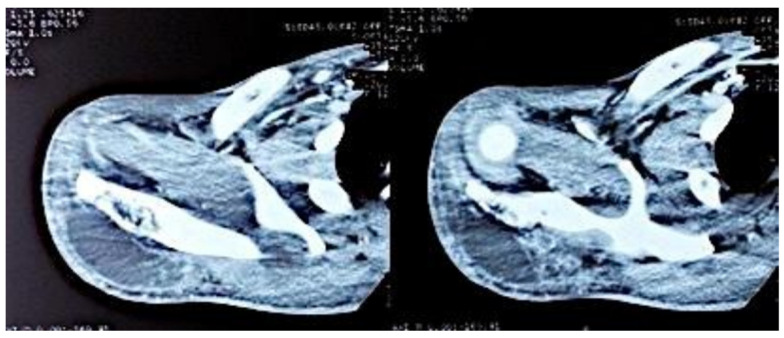
Axial CT sections of the shoulder, showing the lytic lesion of the distal ½ of the right scapular spine.

**Figure 4:**
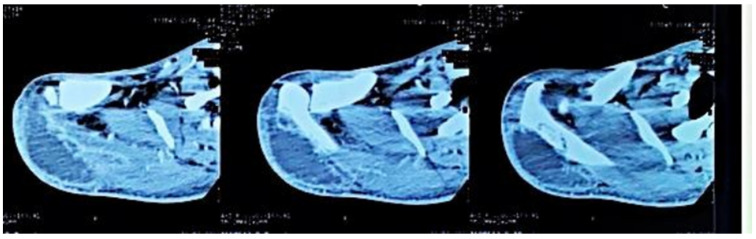
Axial sections of the shoulder CT scan, showing the deltoid collection.

**Figure 5:**
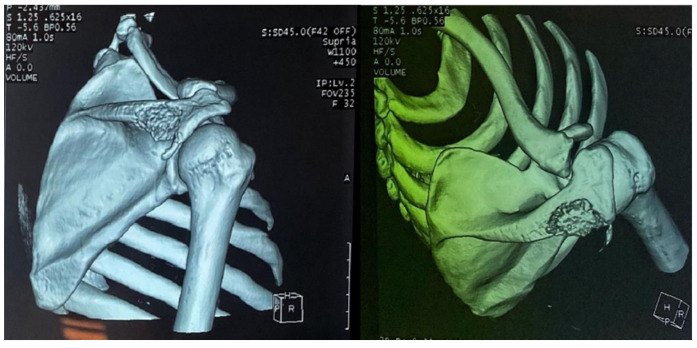
A 3D reconstruction of the right shoulder joint, showing the lytic lesion of the scapular spine.

Computed tomography of the right shoulder showed a lytic lesion of the right scapula with cortical rupture associated with scapular osteomyelitis (Figure 3, Figure 4 and Figure 5). The lesion was complicated by a deltoid abscess measuring 75 × 29 mm and extending over 105 mm (Figure 4), without joint effusion.

The abscess was punctured, and purulent fluid was obtained. No bacteria were found on direct examination, and the culture was sterile. However, Ziehl–Neelsen staining showed the presence of acid-alcohol-resistant bacilli, and the MTB/RIF expert gene test was positive. No rifampicin resistance was detected.

Similarly, the MTB/RIF expert gene test was positive in his sputum, and the LF-LAM urine test was positive, showing grade IV urine.

The remaining laboratory tests showed leukopenia at 2810/µL, normocytic normochromic anemia at 8.5 g/dL, regenerative lymphopenia at 150/µL and C-reactive protein (CRP) at 122 mg/L. HIV serology was positive, confirmed by a viral load of 90,700 copies/mL with profound immunosuppression: CD4 at 9 cells/mm^3^, a CD4 percentage of 6% and a CD4/CD8 ratio of 0.15.

The remainder of sexually transmitted infection (STI) screening was conducted and revealed negative results, including tests for syphilis serology and hepatitis B.

Based on these clinical and biological criteria, a diagnosis of retroviral infection at the AIDS stage was established.

**Figure 6:**
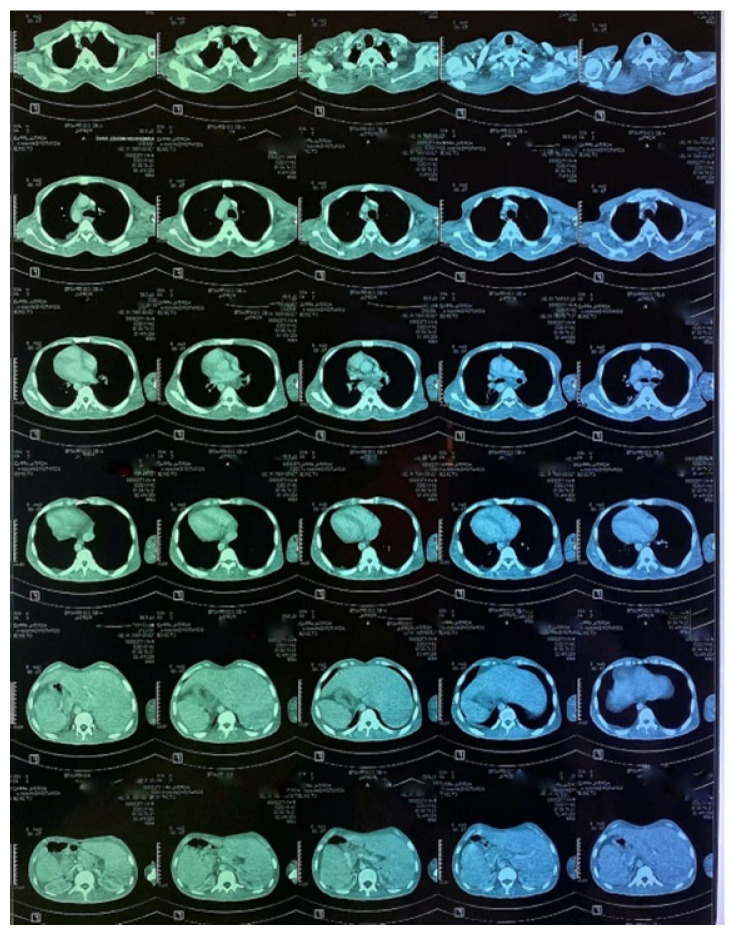
CT images of the thorax and abdomen:axial view.

**Figure 7:**
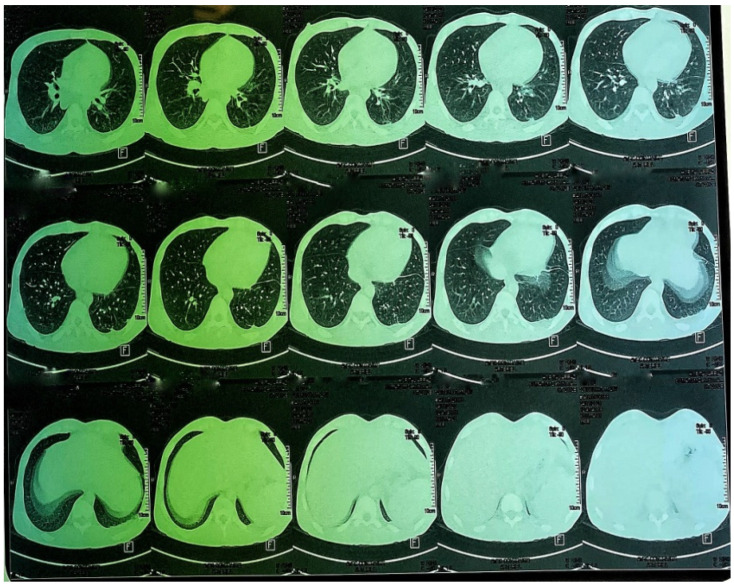
CT images of the thorax and abdomen.

A CT scan of the chest, abdomen and pelvis was subsequently performed, showing a mild left pleural and abdominal effusion associated with right supra-diaphragmatic adenopathy and homogeneous hepatosplenomegaly (Figure 6 and Figure 7).

The patient underwent surgical drainage of the abscess and genotyping in the presence of PrEP, which showed no resistance to the different classes of antiretroviral drugs; especially NRTI resistance or any other potential hidden mutations.

The established diagnosis was a right scapular osteomyelitis complicated by a deltoid abscess associated with pulmonary and abdominal tuberculosis.

The patient was treated using anti-tubercular drugs according to Moroccan recommendations [6]: 2 months of rifampicin-isoniazid-pyrazinamide-ethambutol followed by 10 months of rifampicin-isoniazid (2RHZE/10RH). Triple antiretroviral therapy based on efavirenz/lamivudine/TDF was started within two weeks of taking the antibacterials.

The outcome was satisfactory, with good clinical improvement, apyrexia and progressive recovery of mobility in the affected shoulder.

## 3. Discussion

Osteoarticular tuberculosis is the fourth extrapulmonary site. Its diagnosis is unproblematic in pulmonary concomitant bacillary localizations but often difficult and delayed in isolated joint involvement [7]. This is a pauci-bacillary form that has recently benefited from advances in molecular biology, which is particularly true for the expert gene MTB/Rif.

Tuberculous osteomyelitis occurs as a result of hematogenous dissemination in the bone marrow. It develops in an area of well-vascularized cancellous bone [1]. Less commonly, it develops in association with cutaneous tuberculosis, bursitis or tuberculous tenosynovitis. The initial granulomatous proliferation spreads, and caseous necrosis with osteolysis develops [1].

The skeletal sites most frequently affected by tuberculosis are the bones of the spine, hip, knee and foot [8].

If no joints are affected, tuberculous osteomyelitis may affect the ribs, metatarsals, metacarpals, breastbone, pelvis, skull and, rarely, the large tubular bones. In our case [8], it is interesting to note the unusual location of the osteomyelitis, the scapular spine.

The typical clinical presentation of tuberculous osteomyelitis is painful, insidious swelling of the affected joint or bone, a nonhealing ulcer and purulent discharge. Articular atrophy and periarticular osteoporosis result from joint stiffness. In our patient, the onset was insidious, presenting as a warm, fluctuating, red swelling in the right glenohumeral joint and opposite the scapular region, without ulceration or purulent discharge.

Clinically, osteoarticular TB can mimic other inflammatory and neoplastic bone lesions such as pyogenic osteomyelitis, fungal infection, multiple myeloma and metastatic disease [8]. For our case, the presence of any other germ was ruled out by pus culture and even multiplex PCR. The radiological features of skeletal TB are not specific. They may vary from lytic lesions and periarticular osteoporosis to bone marrow oedema, joint effusion, tenosynovitis and soft tissue collections [9], as in our patient, in whom a CT scan of the shoulder revealed bone lysis of the ½ distal scapular spine with cortical rupture and deltoid collection.

Pauci-bacillary cases of osteoarticular tuberculosis challenge diagnoses because cultured specimens are not sufficiently positive [8].

Another disadvantage of conventional culture is the long incubation period of 6 to 8 weeks [8]. In contrast, our patient had positive results from all the required tests (direct examination and culture for BK; MTB/RIF expert gene in the puncture fluid, sputum and urine).

Treatment for this condition is typically conservative and involves the use of anti-bacillary drugs. In some cases, surgery may be necessary, particularly if complications arise [5]. In our patient, a combination of medical treatment with antibacterial drugs (2RHZE/10RH) and emergency surgical drainage due to the size of the deltoid collection was considered.

## 4. Conclusions

Tuberculous osteomyelitis is a rare form of tuberculosis. Osteomyelitis of the scapular spine is an exceptional form.

The diagnosis of tuberculosis should be made when osteomyelitis or osteoarthritis presents with an insidious onset and a sub-acute or chronic course. HIV serology should be requested systematically in the presence of any tuberculosis infection.

## Data Availability

The original contributions presented in the study are included in the article, further inquiries can be directed to the corresponding author.
